# Incidence and risk factors for pulmonary hemorrhage after percutaneous CT-guided pulmonary nodule biopsy: an observational study

**DOI:** 10.1038/s41598-024-58045-3

**Published:** 2024-03-28

**Authors:** Chuang He, Ling Zhao, Hua-long Yu, Wei Zhao, Dong Li, Guo-dong Li, Hao Wang, Bin Huo, Qi-ming Huang, Bai-wu Liang, Rong Ding, Zhe Wang, Chen Liu, Liang-yu Deng, Jun-ru Xiong, Xue-quan Huang

**Affiliations:** 1 Department of Nuclear Medicine (Treatment Center of Minimally Invasive Intervention and Radioactive Particles), First Affiliated Hospital, Army Medical University, No. 30 of Gao Tanyan District, Chongqing, China; 2 Department of Minimally Invasive Interventional Medicine, Yunnan Cancer Hospital, Yunnan, China; 3 Department of Radiology, Affiliated Hospital of Qingdao University, Shandong, China; 4 Department of Computer Tomograph, Baoshan People’s Hospital, Yunnan, China; 5 Treatment Center of Imaging Minimally Invasive, Beijing Jingxi Cancer Hospital, Beijing, China; 6 Department of Thoracic Surgery, Shanghai Cancer Center of Fudan University, Shanghai, China; 7 Department of Interventional, Affiliated Zhongshan Hospital of Dalian University, Liaoning, China; 8 Department of Oncology, The Second Hospital of Tianjin Medical University, Tianjin, China; 9 Department of Radiology, Second Affiliated Hospital of Fujian Medical University, Fujian, China; 10 Department of Oncology, Dazhou Integrated TCM and Western Medicine Hospital, Sichuan, China; 11 Department of Interventional Therapy, Beijing Cancer Hospital, No. 52 Fucheng Road, Haidian District, Beijing, 100142 China

**Keywords:** Cancer, Lung cancer

## Abstract

To evaluate the current incidence of pulmonary hemorrhage and the potential factors contributing to its increased risk after percutaneous CT-guided pulmonary nodule biopsy and to summarize the technical recommendations for its treatment. In this observational study, patient data were collected from ten medical centers from April 2021 to April 2022. The incidence of pulmonary hemorrhage was as follows: grade 0, 36.1% (214/593); grade 1, 36.8% (218/593); grade 2, 18.9% (112/593); grade 3, 3.5% (21/593); and grade 4, 4.7% (28/593). High-grade hemorrhage (HGH) occurred in 27.2% (161/593) of the patients. The use of preoperative breathing exercises (PBE, p =0.000), semiautomatic cutting needles (SCN, p = 0.004), immediate contrast enhancement (ICE, p =0.021), and the coaxial technique (CoT, p = 0.000) were found to be protective factors for HGH. A greater length of puncture (p =0.021), the presence of hilar nodules (p = 0.001), the presence of intermediate nodules (p = 0.026), a main pulmonary artery diameter (mPAD) larger than 29 mm (p = 0.015), and a small nodule size (p = 0.014) were risk factors for high-grade hemorrhage. The area under the curve (AUC) was 0.783. These findings contribute to a deeper understanding of the risks associated with percutaneous CT-guided pulmonary nodule biopsy and provide valuable insights for developing strategies to minimize pulmonary hemorrhage.

## Introduction

In recent years, the applications of low-dose CT and artificial intelligence have improved the accuracy and sensitivity in detecting pulmonary nodules, most of which are benign^1,2^. Radiologically, these nodules are often classified into three categories: pure ground glass, part-solid and solid nodules. Percutaneous CT-guided pulmonary nodule biopsy (PCPNB) has high accuracy in detecting pulmonary nodules, even subsolid nodules, and has been used for many years to assess and guide the management of pulmonary nodules. It is recommended to evaluate the persistence of subsolid lesions before considering PCPNB^3^. The pooled sensitivity and specificity of PCPNB for identifying subsolid nodules are 90% and 99%, respectively, and core needle biopsy has a marginally greater sensitivity than fine-needle aspiration (93% vs. 83%, p = 0.07)^4^.

According to several guidelines, a growing pulmonary nodular lesion is one of the main indications for PCPNB^5–8^. The risk of complications of PCPNB, such as pneumothorax, pulmonary hemorrhage or other rare complications, can reduce the thoracic surgeon’s confidence in deciding to perform a biopsy^9,10^. Pulmonary hemorrhage is mostly caused by penetrating trauma or the cutting of a bronchial vascular structure^11^. Patients with high-grade hemorrhage (HGH) are more prone to hemoptysis, asphyxia, and infection. HGH can be prevented by reducing the incidence of intrapulmonary hemorrhage, but techniques to do so differ across patients, procedures, biopsy instruments, and characteristics of the pulmonary lesions^8^. Therefore, the benefits and risks of PCPNB for patients with pulmonary nodules need to be assessed separately.

Age, lesion depth, emphysema, nodule size, main pulmonary artery diameter (mPAD) enlargement^9,12–14^, and other factors^5,6,8,9^ have been objectively associated with the development of pulmonary hemorrhage. Moreover, other technical aspects need further exploration, and recommendations for a comprehensive technical solution to reduce the incidence of HGH are needed.

Our primary objectives were to (1) assess the incidence of pulmonary hemorrhage and the possible factors contributing to its increased incidence after PCPNB and (2) summarize the technical solutions available to reduce the occurrence of HGH.

## Materials and methods

This study was approved by the Ethics Committee of the First Affiliated Hospital of the Army Medical University (IRB No. KY2021084). Informed consent was obtained from the patients. The study has also been registered in the Chinese Clinical Trials (ChiCTR2100044574). All the methods described in this manuscript were carried out in accordance with the relevant institutional guidelines and regulations of the Committee of the Chinese Society of Interventional Oncology, China Anti-Cancer Association^6^.

### Study population

**Figure 1:**
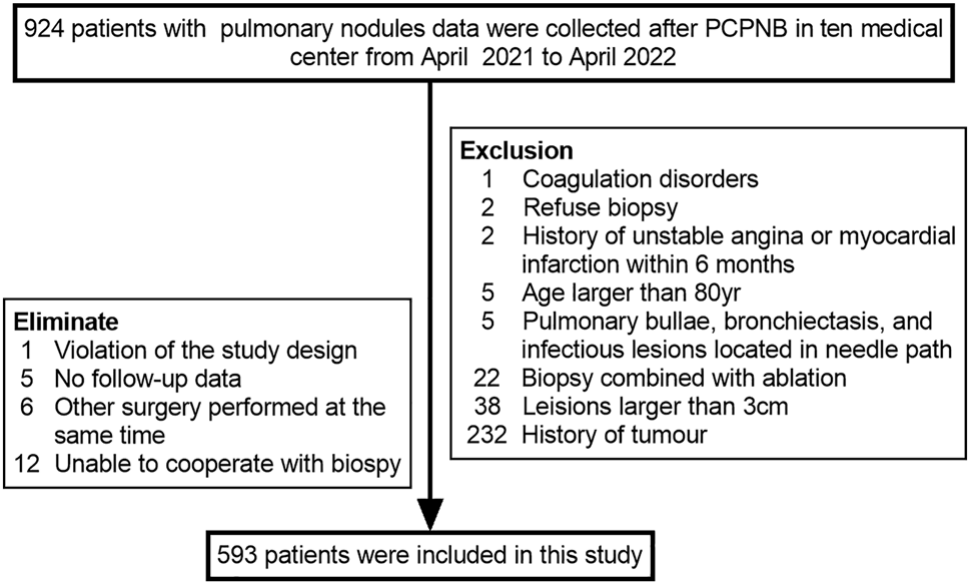
Flowchart showing the inclusion and exclusion criteria for 593 patients who underwent percutaneous CT-guided pulmonary nodule biopsy.

In this prospective study, the data of patients with pulmonary nodules were obtained from ten medical centers from April 2021 to April 2022. Clinical data, including imaging, biopsy procedure information and complication information, were collected from patients who underwent PCPNB and put into an electronic data capture system. The data of 924 patients were recorded, and 593 patients were included in the analysis after the inclusion and exclusion criteria were applied (Fig. 1).

### Indicators and definitions

The primary characteristics included inpatient or outpatient status, age, multidisciplinary treatment (yes vs. no), sex, smoking status (yes vs. no), diabetes status (yes vs. no), and hypertension (> 140/90 mmHg vs. ≤ 140/90 mmHg). The authors categorized the mPAD as less than 29 mm or 29 mm or more on CT images, which has a specificity of 100% in the diagnosis of pulmonary hypertension^15^.

The lesion factor included the maximum diameter of the target lesion on the standard lung window CT axial view (window plane = −600 Hounsfield units, window width = 1500) to determine the size of the lesion. The lobular location of the lung nodules was classified into two categories: the upper lobe (including the upper lobe and right middle lobe) and the lower lobe. The lung nodules were further classified into three categories based on the location of the lesion. Peripheral nodules were defined as lesions located < 2 cm from the parietal pleura, hilar nodules were defined as lesions located < 2 cm from the hilum, and intermediate nodules were defined as lesions located between the peripheral and hilar nodules. The consolidation tumor ratio (CTR) was calculated as the ratio of the maximum diameter of consolidation in the transverse section of the nodule to the maximum diameter of the nodule in the lung window image. The CTR was classified as ≤ 50% or > 50%^16^.

The technical factors include the use of the coaxial or noncoaxial technique, the use of semiautomatic or automatic needles, the use of three-dimensional reconstruction (yes or no), and the use of breathing exercises (yes or no). The patients started practicing the breathing exercises the day before the procedure. The breathing exercise involved the patient taking rhythmic breaths at 16–20 times per minute and then holding their breath for 3–5 s at the end of inspiration, with the goal of keeping the amplitude of each breath constant and the range of chest movement at 2–3 cm. The number of cuts and whether prophylactic hemostatic medication was used (yes or no) were reported. The operation time was defined as the time from scanning the first image to the time of scanning the last image. Depending on the location of the nodule, the patient was operated on in the supine, prone (right or left), or lateral position to ensure the safest and shortest path to the lesion; this was not always the straightest path to the lesion. The length of the puncture needle was measured in terms of the distance from the pleura to the lesion. The needles varied in diameter from 19 to 16 g and were divided into two groups: 16–17 G and 18–19 G. The distribution of the different needle types was as follows: 16G accounted for 29.5% (175/593), 17G accounted for 41% (243/593), 18G accounted for 24.8% (147/593), and 19G accounted for 4.7% (28/593). The passage of the needle through the visceral pleura or oblique fissure was defined as one or two pleural passes, respectively. Immediate contrast-enhanced CT (ICECT) was performed while the patients were in the operative position at the beginning of the procedures. Preoperative contrast-enhanced CT (PCECT) and noncontrast-enhanced CT were also used. The range of CT scans included the surgical area or the whole lung. Patients were asked to perform rhythmic breathing exercises preoperatively to reduce pain and the impact of changes in surgical position on the rhythm of breathing movements.

### Definition of pulmonary hemorrhage

Pulmonary hemorrhage was defined as new consolidative or ground-glass opacities in the perilesional, perineedle path, or other lung lobes on postbiopsy images. Pulmonary hemorrhage was categorized according to a grading scheme modified from a previous hemorrhage grading scheme^9^. Grade 0 was defined as no pulmonary hemorrhage; grade 1 was defined as hemorrhage less than or equal to 2 cm around the needle or lesion; grade 2 was defined as hemorrhage measuring more than 2 cm and less than 4 cm; grade 3 was defined as hemorrhage more than 4 cm; and grade 4 was defined as hemoptysis or bleeding seeping into the other lung lobes.

In this study, we considered Grade 2 or higher hemorrhages to be HGH because the hemorrhage can spread to multiple lobes and increase the risk of infection, and hemoptysis may increase the risk of biopsy failure and asphyxia.

### Statistical analysis

All the data are presented as the means ± standard deviations for continuous variables and as numerical values (percentages) for categorical variables, whereas the medians (interquartile ranges) are presented for nonnormal data. The data were tested for a normal distribution using the Shapiro‒Wilk test. The data were subjected to univariate analysis using t tests for continuous variables and Pearson's χ^2^ test, or Fisher's exact test for categorical variables for comparison. A logistic regression with multiple explanatory variables was used for variable selection. At the very beginning, all variables believed to be potentially related to the outcome were included in the model. Then a backward stepwise selection procedure based on likelihood ratio test was performed. The final selected variables were those that remained statistically significant (p < 0.05) after the stepwise procedure. All reported P values are two-sided and a p value < 0.05 was considered statistically significant. The statistical analysis was performed using SPSS Statistics (version 26, IBM.)

## Results

A total of 593 patients were included in this study (Table 1), 31.7% (188/593) of whom were outpatients and 13.8% (82/593) of the patients underwent percutaneous puncture biopsy after a multidisciplinary team (MDT) consultation was conducted. The procedure was performed in 97.1% (576/593) of patients in a state of free breathing. Eighteen percent (107/593) of patients performed breathing exercises before PCPNB. Patients with diabetes accounted for 10.6% (64/593), and 39.5% (234/593) of patients had hypertension (140/90 mmHg). The incidences of pulmonary hemorrhage were as follows: grade 0, 36.1% (214/593); grade 1, 36.8% (218/593); grade 2, 18.9% (112/593); grade 3, 3.5% (21/593); and grade 4, 4.7% (28/593). HGH occurred in 27.2% (161/593) of the patients.

**Table 1:** Patient, nodule, and technical characteristics related to low- and high-grade pulmonary hemorrhage.

Variable	Pulmonary hemorrhage				*P*
	Low grade (N = 432)		High grade (N = 161)		
Patient					
Hospitalization					0.849
Outpatient	136		52		
Inpatient	296		109		
MDT					0.205
No	377		134		
Yes	55		27		
Age (years)	59.43 ± 11.17		58.78 ± 11.66		0.529
Sex					0.234
Male	225		75		
Female	207		86		
BMI	23.4 ± 3.27		23.27 ± 2.75		0.679
Smoker					0.068
No	274		115		
Yes	158		46		
Diabetes					0.219
No	382		148		
Yes	50		13		
Hypertension					0.929
No	262		97		
Yes	170		64		
Emphysema					0.202
No	369		144		
Yes	63		17		
mPAD (mm)					0.433
< 29	271		94		
≥ 29	102		42		
Nodule					
Size (cm)	1.87 ± 0.68		1.77 ± 0.69		0.105
Lobe					0.652
Upper	229		82		
Lower	203		79		
Location					0.022
Peripheral	337		108		
Hilar	33		17		
Intermediate	62		36		
CTR					0.293
≤ 50%	95		42		
> 50%	337		119		
Cavitary					0.616
Yes	15		7		
No	417		154		
Technical					
Breathing training					0.000
No	338		148		
Yes	94		13		
Operation time	17.30 ± 9.59		15.69 ± 8.71		0.059
Position					0.926
Supine	174		62		
Prone	172		66		
Lateral position	86		33		
Contrast-enhanced CT					0.008
No	124		50		
ICECT	201		54		
PCECT	107		57		
Visceral pleural passes					0.035
1	368		123		
2	53		33		
3	11		5		
Puncture length	3.47 ± 2.41		4.07 ± 2.43		0.007
Reconstruction					0.878
No	404		150		
Yes	28		11		
Needle size					0.000
16 or 17 G	327		91		
18 or 20 G	105		70		
Mode of cutting					0.383
Semiautomatic	248		86		
Automatic	184		75		
Coaxial					0.000
Coaxial	225		46		
Noncoaxial	207		115		
Range of CT					0.542
Regional	181		63		
Whole	251		98		
Prophylactic medication					0.000
Yes	50		42		
No	382		119		
Number of cuttings	1.75 ± 0.93		1.49 ± 0.69		0.001

*MDT* multidisciplinary treatment, *ICECT* immediate contrast-enhanced CT, *PCECT* preoperative contrast-enhanced CT.

Hemoptysis occurred in 2.8% (17/593) of the patients after biopsy, and the median hemoptysis volume was 10 mL (IQR 3.5, 100). However, only 6.5% (39/593) of the patients with hemorrhage underwent medical intervention. Air embolism, a rare complication, occurred in 0.2% (1/593) of the patients in the HGH group. Hemothorax occurred in 0.35% (21/593) of the patients who underwent biopsy.

### Univariate analysis of patients with high-grade hemorrhage

Univariate analysis (Table 1) demonstrated that the occurrence of high-grade hemorrhage was significantly associated with location (p = 0.022), breathing exercises (p = 0.000), contrast-enhanced CT (p = 0.008), number of pleural passes (p = 0.012), puncture length (p = 0.007), needle size (p = 0.000), coaxial technique (p = 0.000), prophylactic medication for hemostasis (p = 0.000), and the number of cuttings (p = 0.000). Other patient-, lesion-, and technique-related variables were not significantly associated with the occurrence of HGH. Based on past procedures and previous literature results^9,12,14,17^, the potential risk factors for pulmonary hemorrhage were as follows: prolonged operation time (p = 0.059), nodule size (p = 0.307), CTR > 50% (p = 0.293), involvement of the upper lobe (p = 0.652), surgical position (p = 0.926), CT reconstruction (p = 0.878), use of semiautomatic needles (p = 0.383), mPAD > 29 mm (p = 0.433), and emphysema (p = 0.202).

### Multivariate analysis of high-grade hemorrhage

**Figure 2:**
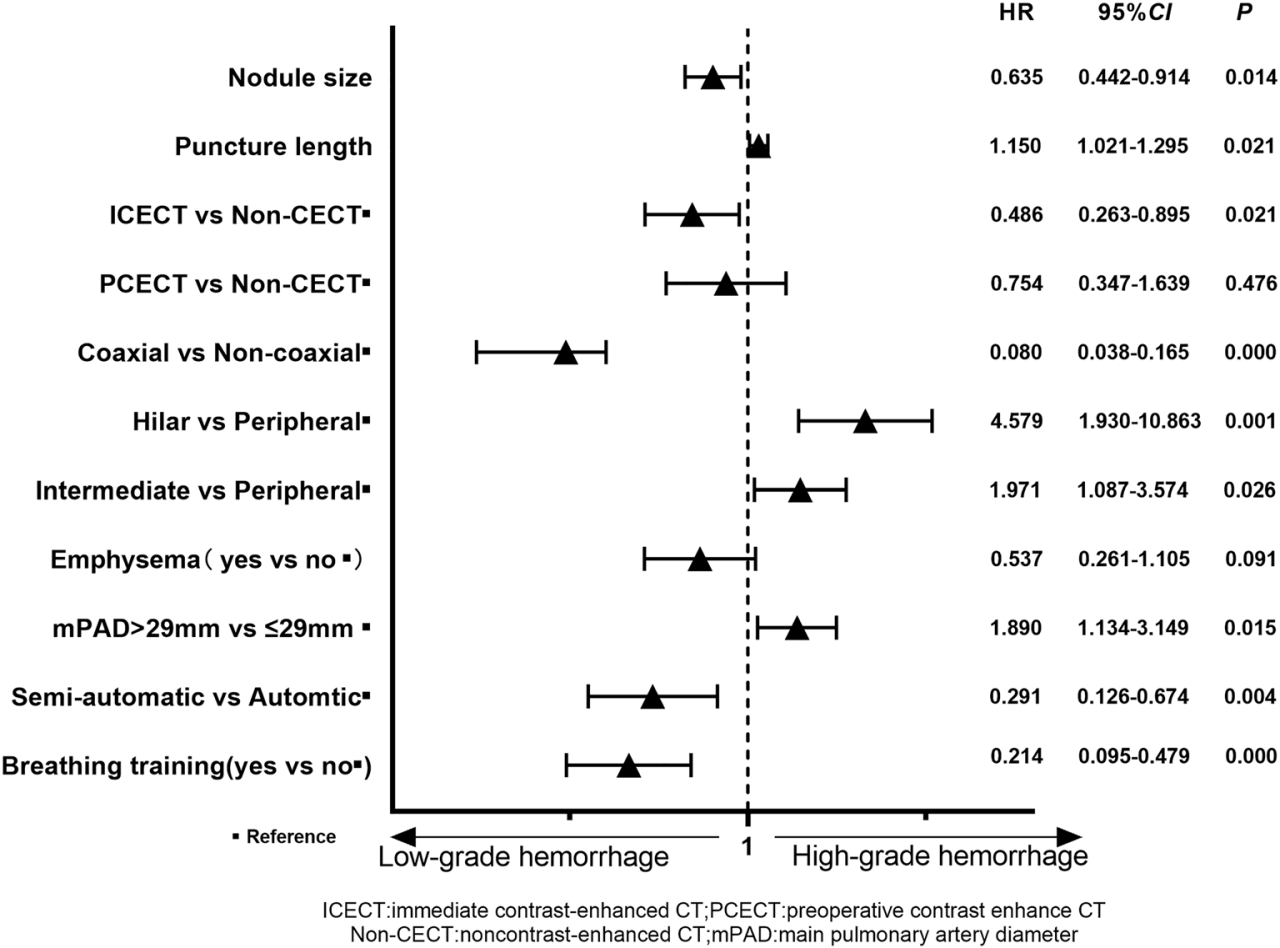
Multivariate analysis (backward: LR) results for patients with HGH. Breathing exercises, semiautomatic cutting needles, immediate contrast-enhanced CT, and coaxial techniques are useful for preventing HGH.

**Figure 3:**
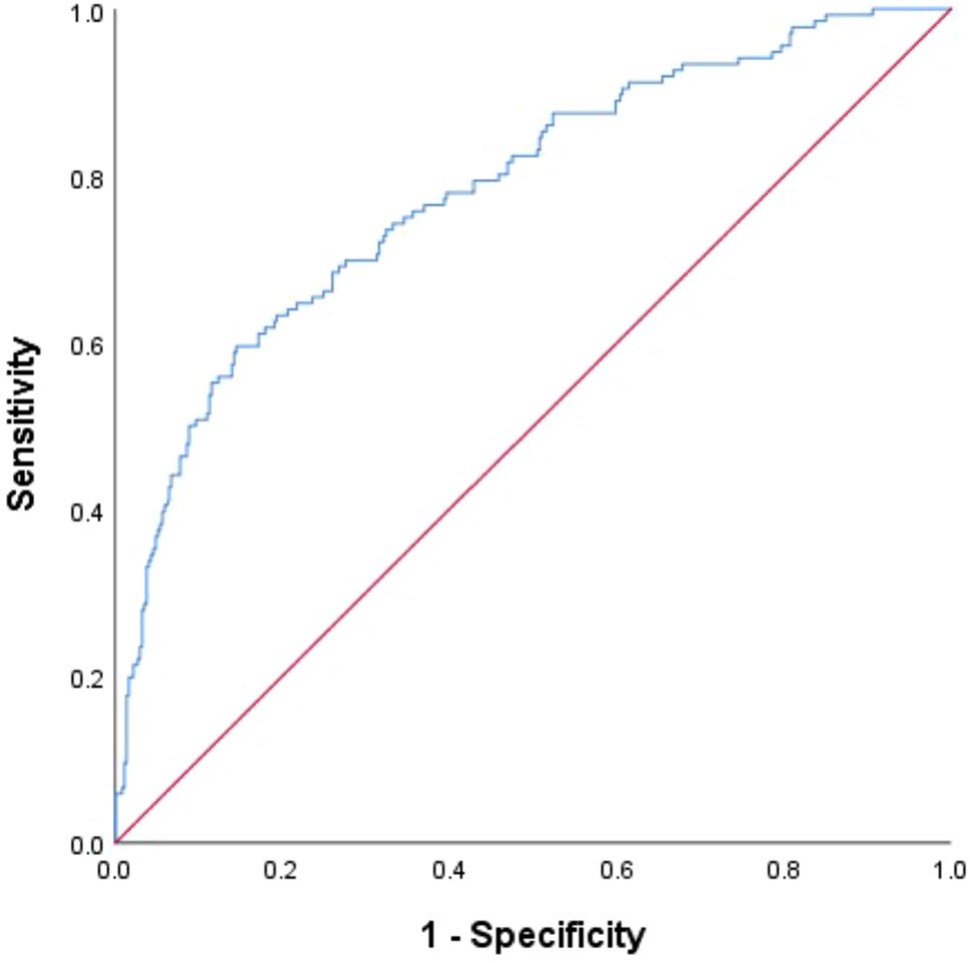
The probability of HGH was calculated for each procedure, and the ROC curve was drawn based on the likelihood. The AUC was 0.783 (95% CI = 0.737–0.829).

The multivariable logistic regression results are shown in Fig. 2. Breathing exercises (HR 0.214, 95% CI 0.095–0.479), use of semiautomatic needles (HR 0.291, 95% CI 0.126–0.674), coaxial technique (HR 0.080, 95% CI 0.038–0.165), and ICECT (HR 0.486, 95% CI 0.263–0.895) were protective factors for HGH, respectively. The risk of HGH was significantly increased in patients with a mPAD > 29 mm (HR 1.890, 95% CI 1.134–3.149), a greater puncture length (HR 1.150, 95% CI 1.021–1.295), hilar nodules (HR 4.579, 95% CI 1.930–10.863), intermediate nodules (HR 1.971, 95% CI 1.087–3.574), or a small nodule size (HR 0.635, 95% CI 0.442–0.914). Other variables were not significantly associated with the occurrence of HGH. These factors had clinical value in predicting HGH, with an AUC of 0.783 (95% CI 0.737–0.829) (Fig. 3).

## Discussion

In this study, pulmonary hemorrhage occurred in 379 of 593 patients (63.9%), and high-grade hemorrhage occurred in 161 (27.2%) patients. The preoperative breathing exercise (PBE)/semiautomatic cutting needle (SCN)/ICE/CoT (PSIC) approach was considered a protective factor against HGH. A greater puncture length, hilar nodules, intermediate nodules, mPAD > 29 mm, and greater nodule size were risk factors for HGH.

The leading causes of pulmonary hemorrhage are penetrating and cutting injuries to bronchovascular structures. Bleeding is usually self-limiting, but deaths caused by massive intrapulmonary hemorrhage have also been reported^18^. The overall hemorrhage rate in the present study was greater than that in a previous study^9,12,13^. However, the need for intervention was uncommon; approximately 6.5% (39/593) of patients in our study underwent medical intervention. Hemoptysis is the direct clinical manifestation of pulmonary hemorrhage and occurs in 2.8% of patients; this incidence is lower than the reported rate of 3.03–11%^9,11,12,14,19–21^ but higher than that reported by Tai. This difference may be partly due to possible underreporting of self-limited hemoptysis in the radiology reports described in Tai's study^9^. In addition, hemothorax is a rare complication of percutaneous transthoracic needle biopsy. It occurred in 0.35% of the patients who underwent biopsy, which is consistent with prior observations (0.20–0.92%)^19^.

The key to preventing pulmonary hemorrhage is to avoid vascular damage along the needle tract and around or inside the nodule. Therefore, first, the characteristics of the nodules need to be objectively assessed, and then certain technical factors need to be evaluated to identify the best method for reducing the occurrence of pulmonary hemorrhage.

In this study, the authors summarize a technical protocol called PSIC, a protective factor for HGH. The following technical aspects should be fully considered despite not being considered in other studies on pulmonary hemorrhage^4,12,17,22^.

First, we found that preoperative breathing exercises significantly reduced the incidence of HGH and were a protective factor against HGH. No retrospective studies have evaluated this simple and feasible preoperative assistive technique. Regular breathing is highly beneficial because operators can avoid injuring the pulmonary vasculature; therefore, most of the procedures that were performed in this study involved patients who were in a steady free breath state. In a randomized study, the complications of CT-guided lung biopsy did not lead to the need for an interactive bellows-based breath-hold control system^23^.

Second, no studies have compared the incidence of pulmonary hemorrhage between semiautomatic and automatic needle use in lung nodule biopsy. The incidence of HGH was lower with semiautomatic cutting needles than with automatic cutting needles in this observational study. Semiautomatic cutting needles are more beneficial because they can avoid vessels around or inside the nodule. It is easier for the operator to control the direction of the tip of the needle entering the lesion and the direction of the sampling slot of the needle. In addition, the impact force from automatic cutting is greater than that from automatic cutting, thus increasing the risk of damaging vessels around the nodule.

Third, the coaxial technique not only reduces the number of pleural passes but can also be used to separate pulmonary vessels, thereby reducing the occurrence of pulmonary hemorrhage. Pulmonary hemorrhage is significantly associated with the number of pleural passes and pass-through vessels^13,17^. However, several studies have shown that the coaxial technique is a risk factor for pulmonary hemorrhage or that there is no significant difference in the incidence of pulmonary hemorrhage between coaxial and noncoaxial processes^9,13^. Pulmonary nodules were not exclusively included in these studies, and the importance of preoperative or immediate contrast-enhanced CT was ignored.

Finally, enhanced thin-slice CT shows the distribution of vessels located around, at the margins, and inside the nodule, thus reducing the risk of considerable bleeding caused by cutting. These results are consistent with those of Hu's^24^ observation that contrast-enhanced CT (ICECT and PCECT) scanning can significantly reduce the risk of postbiopsy bleeding. However, when we classified ICECT and PCECT images in this study, we found that ICECT was a protective factor against HGH and that PCECT was not associated with a significantly lower risk of HGH than was noncontrast-enhanced CT. Therefore, a safer puncture path should be designed, and biopsy should be performed under ICECT.

Additional patient-related variables that were found to be significantly associated with high-grade hemorrhage include mPAD, nodule size, and nodule location.

In clinical practice, it has been reported that pulmonary artery pressure should not be measured by right-sided heart catheterization in all PCPNB patients; therefore, we indirectly measured the mPAD on CT^9,14,15^. In our study, mPAD was identified as a risk factor for HGH, as confirmed by previous studies^14,25^, but another study showed that a large mPAD was not a risk factor for HGH^9^. Moreover, a ratio of mPAD to aortic diameter greater than 1 is a risk factor for HGH^12^. Nevertheless, the association between mPAD and pulmonary hypertension has been debated in the literature^9^. Therefore, we can apply the mPAD results of this study in the clinical application of pulmonary nodule biopsy until higher-level evidence is obtained.

We categorized pulmonary nodule location for the first time. We found that nodules located in the hilar and intermediate regions were at the highest risk for HGH due to their deep location, long puncture path, and number of vessels located around or passing through the pulmonary nodules. A greater puncture length was considered a high risk factor for HGH. The design of the puncture path should fully consider technical and objective factors. Previous studies have confirmed that a long needle length is positively associated with the risk of pulmonary hemorrhage^13,17,21^ and that the smaller the nodule is, the greater the risk of bleeding^9,12,14,16,17,24,26^, which is consistent with our study.

Therefore, in the presence of these objective factors, it is necessary to compensate for these risk factors by designing a reasonable puncture path and considering the technical factors that were described in this study to avoid damaging the vessels in the puncture path and to prevent bleeding caused by cutting the biopsy needle.

This study has several limitations. First, because this was a prospective real-world observational study in which data were collected from multiple centers, no patient randomization was performed, as in randomized clinical trials. Second, we classified pulmonary nodules according to radiologic morphology, but the results of CTR assessments may differ across centers. Our study used 50% as the cutoff point and found that the difference in pulmonary hemorrhage incidence was statistically significant (p = 0.000); however, the incidence of HGH was not significantly different between the two groups (p = 0.293), which is partly consistent with the results obtained by Yun et al.^22^. Third, we divided needle sizes into only 16–17 G and 18–19 G, and the complications of various types of needles were not compared; moreover, some studies have not shown a significant difference in the risk of hemoptysis between 17 and 20 G and 22 G needles^19^. Additionally, there was no considerable difference in complication rates between the fine-needle aspiration and core needle biopsy groups^4^. Furthermore, as this study is still ongoing, we are currently unable to provide conclusive data on the diagnostic accuracy of percutaneous biopsy for pulmonary nodules. Finally, further studies are needed to confirm the effect of nodule morphology and needle size on the incidence of pulmonary hemorrhage.

## Conclusion

Pulmonary hemorrhage commonly occurs after PCPNB and rarely requires intervention. In this study, the authors provide a summary of the PSIC protocol, which has been found to have a protective effect on HGH after PCPNB. Additionally, the protocol has potential benefits for the development of puncture instruments specific for pulmonary nodule biopsy. Moreover, a mPAD > 29 mm, a greater length of puncture, the presence of hilar nodules, the presence of intermediate nodules, and a small nodule size were significantly associated with high-grade hemorrhage. However, further verification through a prospective randomized controlled trial is necessary to confirm these results.

## Data Availability

The datasets used and/or analyzed during the current study are available from the corresponding author upon reasonable request.
